# Comparison and Advanced Antimicrobial Strategies of Silver and Copper Nanodrug-Loaded Glass Ionomer Cement against Dental Caries Microbes

**DOI:** 10.3390/antibiotics11060756

**Published:** 2022-06-02

**Authors:** Amal Adnan Ashour, Mohammed Fareed Felemban, Nayef H. Felemban, Enas T. Enan, Sakeenabi Basha, Mohamed M. Hassan, Sanaa M. F. Gad El-Rab

**Affiliations:** 1 Department of Oral and Maxillofacial Surgery and Diagnostic Sciences, Oral Pathology Division, Faculty of Dentistry, Taif University, Taif 26571, Saudi Arabia; a.a.ashour@tu.edu.sa; 2 Department of Oral and Maxillofacial Surgery and Diagnostic Sciences, Periodontics Division, Faculty of Dentistry, Taif University, Taif 26571, Saudi Arabia; mfelemban@tudent.edu.sa; 3 Preventive Dentistry Department, Faculty of Dentistry, Taif University, Taif 26571, Saudi Arabia; nfelemban@tudent.edu.sa; 4 Department of Dental Biomaterials, Faculty of Dentistry, Mansoura University, Mansoura 35511, Egypt; enasenan275@mans.edu.eg; 5 Department of Preventive and Community Dentistry, Faculty of Dentistry, Taif University, Taif 26571, Saudi Arabia; sakeena@tudent.edu.sa; 6 Department of Biology, College of Science, Taif University, P.O. Box 11099, Taif 21944, Saudi Arabia; m.khyate@tu.edu.sa; 7 Department of Botany and Microbiology, Faculty of Science, Assiut University, Assiut 71516, Egypt

**Keywords:** antimicrobial efficiency, AgNPs, caries lesions, GIC, metronidazole, TVE-CuNPs

## Abstract

Caries lesions during cement repairs are a severe issue, and developing a unique antimicrobial restorative biomaterial can help to reduce necrotic lesion recurrence. As a result, *Thymus vulgaris* extract was used to biosynthesize copper nanoparticles (TVE-CuNPs) exhibiting different characteristics (TVE). Along with TVE-CuNPs, commercial silver nanoparticles (AgNPs) and metronidazole were combined with glass ionomer cement (GIC) to test its antibacterial efficacy and compressive strength. FTIR, XRD, UV-Vis spectrophotometry, and TEM were applied to characterize the TVE-CuNPs. Additionally, AgNPs and TVE-CuNPs were also combined with metronidazole and GIC. The modified GIC samples were divided into six groups, where groups 1 and 2 included conventional GIC and GIC with 1.5% metromidazole, respectively; group 3 had GIC with 0.5% TVE-CuNPs, while group 4 had 0.5% TVE-CuNPs with metronidazole in 1.5%; group 5 had GIC with 0.5% AgNPs, and group 6 had 0.5% AgNPs with metronidazole at 1.5%. An antimicrobial test was performed against *Staphylococcus aureus* (*S. aureus*) and *Streptococcus mutans* (*S. mutans*) by the disc diffusion method and the modified direct contact test (MDCT). GIC groups 4 and 6 demonstrated a greater antimicrobial efficiency against the two tested strains than the other groups. In GIC groups 4 and 6, the combination of GIC with two antimicrobial agents, 1.5% metronidazole and 0.5% TVE-CuNPs or AgNPs, enhanced the antimicrobial efficiency when compared to that of the other groups with or without a single agent. GIC group specimens combined with nanosilver and nanocopper had similar mean compressive strengths when compared to the other GIC groups. Finally, the better antimicrobial efficacy of GIC boosted by metronidazole and the tested nanoparticles against the tested strains may be relevant for the future creation of more efficient and modified restorations to reduce dental caries lesions.

## 1. Introduction

Dental caries, one of the famous common chronic oral diseases in humans worldwide [1,2], is related to a wide range of Gram-positive and Gram-negative microorganisms. According to the contemporary caries etiology hypothesis, an imbalance of oral flora can cause acid accumulation and tooth demineralization, resulting in caries formation. According to the World Health Organization, dental caries affects roughly three-quarters of the world’s population [3]. Caries treatment is still dependent on filling repair at the moment. Therefore, a hotspot in the field of caries suppression and treatment is the evolution of novel anti-caries materials. Metals such as silver, gold, copper, zinc, titanium, and others have been used as antimicrobial agents for centuries to solve this problem [4]. Each metal has unique properties and activity spectra. Because of their delivery capabilities, biocidal, and anti-adhesive properties, silver and copper nanoparticles have received the most attention in recent years [5]. In comparison to silver nanoparticles, few studies have reported copper’s antimicrobial properties against oral microbes, and copper nanoparticles bind to SH groups and disrupt bacterial nucleic acids and key enzymes. The copper nanoparticles have a high thermal and electrical conductivity and are less expensive than silver nanoparticles [6]. The metal nanoparticles can be synthesized by biological methods using plants. Typically, *Thymus vulgaris* leaf oil or extract is used to treat sore throats, tonsillitis, gum disease, rheumatism, and arthritis [7,8,9] and can also be used in the biosynthesis of nanoparticles. AgNPs kill bacteria by a variety of mechanisms, including the rupture of the bacterial cell membrane via AgNP adherence and the penetration of AgNPs into the cell and nucleus, resulting in binding interactions with proteins and DNA and, ultimately, cell death [10]. Combinations of AgNPs with antibiotics have been reported to have synergistic antibacterial effects toward both nonresistant and resistant strains [11]. According to Xu et al. [12], nanoparticles such as those of silver or copper have good mechanical properties, allowing them to be used in dental restorative cements. Glass ionic cement (GIC) is a popular restorative material in dentistry due to its biocompatibility, antimicrobial action, adhesion to dental structures, and fluoride release [13]. The GIC surface erodes because of biofilm formation [14]. To solve this problem, restorative materials have been mixed with antibiotics in previous studies because they have antimicrobial properties [15]. Nonetheless, due to the emergence of multidrug resistant pathogens, traditional antimicrobials are becoming ineffective in treating oral infections. Metronidazole is one of these antibiotics that has antimicrobial activity and inhibits DNA synthesis in anaerobic microbes [16]. Metronidazole resistance has been described in dental abscesses and anaerobic streptococcus illness [17].

To the best of the authors’ knowledge, the effect of antibiotic (metronidazole), AgNPs, and biosynthesized CuNPs in novel GIC biomaterial formulations against oral microbes has never been studied. This study uses *Thymus vulgaris*, which is used in dental applications, to produce TVE-CuNPs. The principal aim of this research is to investigate the antimicrobial efficacy of GIC after being treated with metronidatole, commercial AgNPs, and TVE-CuNPs to bio-fabricate a novel anti-microbial dental biomaterial. The researchers tested modified GIC as a novel anti-microbial dental filling biomaterial against caries-causing oral microbes. This research is significant because improving the antibacterial efficiency and compressive strength of GIC dental cements will result in modified GIC cements with superior properties that can increase the longevity of the restorations and reduce recurrent caries.

## 2. Materials and Methods

### 2.1. Experimental Design

**Scheme 1:**
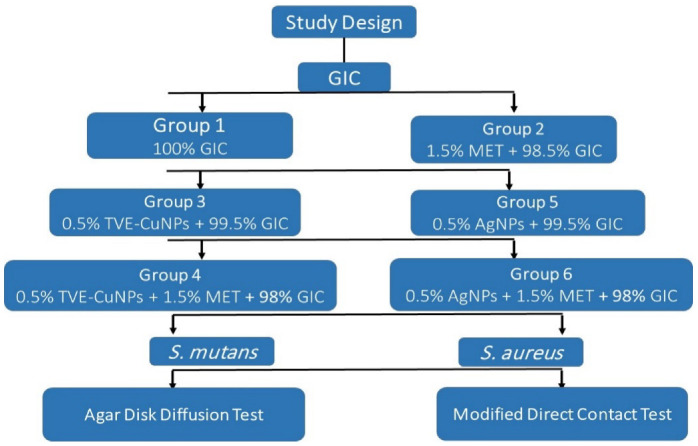
Outline of the study design of conventional GIC and modified GIC against *S. mutans* and *S. aureus*. MET = Metronidazole.

This study used GIC. GIC was mixed with three antimicrobial agents: metronidazole, AgNPs, and TVE-CuNPs. Commercially available AgNPs powder of size 20 to 50 nm (Alibaba Company, Shanghai Xinglu Chemical Technology Co., LTD, Shanghai, China) was purchased. The antimicrobial efficiency of the modified dental cement on two common microbial strains, *S. mutans* and *S. aureus*, was determined using Kirby–Bauer agar diffusion and modified direct contact tests (Scheme 1).

### 2.2. Materials and Equipment

The GIC (GC Fuji IX, Tokyo, Japan) was used in this study, as well as three antimicrobial agents: commercial AgNPs (Sigma-Aldrich, Saint Louis, MO, USA), TVE-CuNPs, and metronidazole (VETRANAL^®^, analytical standard, Sigma-Aldrich, Saint Louis, MO, USA). Thymus vulgaris L extract was used to biosynthesize TVE-CuNPs. Before manipulation, the AgNPs, TVE-CuNPs, and metronidazole were weighed and mixed with GIC powder.

### 2.3. Biosynthesis of Copper Nanoparticles

*Thymus vulgaris* (25 g) was added to 100 mL of distilled water. The solution was heated at 80 °C for one hour to prepare an aqueous extract, as shown in our previous study by Gad El-Rab et al. [18]. In brief, 40 mL of copper chloride (2 mM) was mixed with 10 mL of Thymus vulgaris extract (TVE) and stirred for more than 2 h at room temperature with constant magnetic stirring (200 rpm). The solution mixture’s color changed from deep brown to yellowish brown during the reaction. It is suggested that TVE-CuNPs were formed.

#### 2.3.1. TVE-CuNP Characterization

The characterization of TVE-CuNPs was performed using the following methods.

##### UV-Visible Spectrum

This analysis was conducted using a UV–Vis spectrometer (Shimadzu UV-1650, Tokyo, Japan) in the range of 300–800 nm to confirm the manufacture of TVE-CuNPs [15]. After that, the final mixture was centrifuged at 10,000× *g* to separate the TVE-CuNPs. Finally, brown-black TVE-CuNPs were recovered.

##### Transmission Electron Microscopy (TEM)

TEM analysis was carried out on TEM JEOL at 100 kV, Tokyo, Japan (Assiut Electron Microscope Unit). Each sample for TEM analysis was prepared by placing a drop of the suspension on carbon-coated copper grids and allowing it to dry on the grid for 4 min. The shape and size of TVE-CuNPs were determined by a TEM micrograph [16].

##### X-ray Diffraction

A thin layer of well-grinded dry TVE-CuNPs was distributed on the glass slide that was introduced into the XRD chamber to study the XRD pattern. The phase of TVE-CuNPs was characterized using Shimadzu XRD (3A, Tokyo, Japan), and the spectra were recorded by CuKα radiation with a wavelength of 1.5406Å in the 2θ (from the range of 20–80°) [15].

##### Fourier Transform Infrared Analysis (FT-IR)

To determine the functional groups that are responsible for TVE-CuNP formation and stabilization [19], as KBr pellets, the FTIR spectra of TVE and TVE-CuNPs were recorded on a Shimadzu IR-470 Spectrometer, Tokyo, Japan, in the range of 4000–500 cm^−1^.

### 2.4. Preparation and Characterization of AgNPs

The AgNPs with diameters of 20–50 nm were obtained from Alibaba Company, Shanghai Xinglu Chemical Technology Co., LTD, Shanghai, China. The samples were re-suspended in deionized water at the concentration of 1 mg/mL. The UV–Vis spectra of the AgNPs were recorded using a UV–Vis spectrometer (Shimadzu UV-1650, Tokyo, Japan). The dimensions and sizes of the AgNPs were confirmed using a transmission electron microscope TEM JEOL at 100 kV (Assiut Electron Microscope Unit).

### 2.5. Preparation of Specimens

A split Teflon mold (3 mm in height and 6 mm in diameter) was used to create disc-shaped specimens of GIC cement (180 disc) [18]. All GIC specimens were prepared at room temperature. The GIC powder and liquid were apportioned and combined using a plastic spatula for 30 s to avoid dehydration of the GICs, as indicated by the manufacturer, before being inserted into the molds. The molds were covered on both sides with polyester strips and thick glass plates and let to set for 20 min before being taken from the mold and sanitized for 30 min with UV light. The modified GIC samples (*n* = 180) were divided into six groups of 30 specimens each, as follows:

1. (Group 1) GIC.
2. (Group 2) GIC and 1.5% metronidazole.
3. (Group 3) 99.5% GIC with 0.5% TVE-CuNPs.
4. (Group 4) 98% GIC with 0.5% TVE-CuNPs and 1.5% metronidazole.
5. (Group 5) 99.5% GIC with 0.5% AgNPs.
6. (Group 6) 98% GIC with 0.5% AgNPs and 1.5% metronidazole.

All groups were tested for antibacterial efficacy against *S. aureus* and *S. mutans* (at 1 day, 1 week, and 1 month).

### 2.6. Drug Release Determination

To determine metronidazole release from modified GIC, the samples were submerged in 5 mL of phosphate-buffered saline (PBS) at pH 7.4 and incubated at 37 °C for up to 30 days. A UV-Vis spectrophotometer (Shimadzu UV-1650 pc spectrophotometer, Tokyo, Japan) was used to quantify the concentration of metronidazole in the release medium at 340 nm using a calibration curve. The metronidazole release (%) was calculated using the equation: C (%) = B/A × 100, where C is the metronidazole release (%), B is the total quantity of metronidazole released in the solution, and A is the amount of loaded metronidazole in modified GIC samples.

### 2.7. Bacteria and Growth Conditions

Strains of *S. aureus* and *S. mutans* were obtained from our previous study by Enan et al. [18]. Before performing the antimicrobial test, fresh inoculums of *S. aureus* and *S. mutans* were inoculated in Mannitol Salt Agar medium and Brain Heart Infusion (BHI) medium (Oxoid Ltd., London, UK), respectively, and adjusted to the famous standard (i.e., 1.5 × 10^8^ CFU/mL) with the help of BHI and Mannitol Salt medium, respectively, and the culture was incubated in growing conditions (18 h at 37 ± 2 °C).

#### 2.7.1. Amicrobial Efficacy Using Agar Disc Diffusion Assay

The disc diffusion assay was followed to determine the inhibition zones of the test modified GIC [19]. Mueller–Hinton agar was prepared and added to the Petri plates upon autoclaving and kept for setting or solidifying for 10–15 min. The targeted test for *S. aureus* and *S. mutans* of 0.1 mL was added to media plates and spread with sterile cotton swabs evenly throughout the plates. The test modified GIC discs were placed on the plates, which were kept in an incubator at 37 °C for growth. The inhibition zones were recorded with the zone interpretation scale. All experiments were run in triplets. The antibacterial efficiency of modified GIC discs was evaluated at three time intervals: 24 h, 1 week, and 1 month.

#### 2.7.2. Modified Direct Contact Test

The modified direct contact test (MDCT) [20] is based on determining the colony-forming unit (CFU) of bacterial growth in 96-well microtiter plates. A microtiter plate was held horizontally, and the floor of the wells was evenly coated with a thin layer of GIC. The side walls of 96-well microtiter plates were coated with a thin layer of GIC. In accordance with the manufacturer’s recommendation, the modified luting cement was allowed to set. A total of 50 µL of *S. aureus* or *S. mutans* (approximately 10^6^) were placed on the GIC with 100 µL of Mueller–Hinton broth or brain–heart infusion broth, respectively, for *S. aureus* or *S. mutans*. A positive control, as prepared by placing 50 µL of bacterial suspension along with 100 µL of Mueller–Hinton broth or brain–heart infusion broth in a separate well without the GIC cement, was considered. After 24 h, the *S. aureus* or *S. mutans* suspension was introduced in Mueller–Hinton agar or brain–heart infusion agar plates for *S. aureus* or *S. mutans*, respectively, and the CFUs of the suspension were compared. The data were recorded approximately 24 h after incubation. Additional tests were performed on a set of modified GIC that had been aged for one month.

### 2.8. Compressive Strength Measurement

GIC samples (4 mm in diameter and 6 mm in height) were produced for the compressive strength test (CS) using a Teflon mold. For 20 min, the GIC samples were left at room temperature. After 24 h of mixing, the CS of GIC cement was measured using a Material Test System (810 MTS Co., Minneapolis, MN, USA) at a crosshead speed of 0.5 mm/min^−1^. Six samples were tested for each GIC sample group. The highest recorded force was measured at the fracture, and CS (N/mm^2^) was calculated using the equation shown below [18].

CS = 4*P*/π*d*^2^ (1)

where *P* denotes the failure load and *d* denotes the sample’s diameter.

### 2.9. Statistical Analysis

The mean difference was calculated using One-way Statistical Analysis of Variance (ANOVA) and Tukey’s Post Hoc test in the Statistical Package for Social Sciences version 17 software (SPSS Inc., Chicago, IL, USA). All statistical tests were two-sided, with a significance level of *p* < 0.05.

## 3. Results

### 3.1. Biosynthesis of TVE-CuNPs

TVE-CuNPs were biosynthesized in the current study by reducing copper chloride to TVE-CuNPs using TVE. A change in color from blue to brown indicates the green-mediated synthesis of TVE-CuNPs in the reaction mixture. The formation of brown color in an aqueous solution of TVE-CuNPs was caused by the excitation of surface plasmon resonance (SPR) [21].

### 3.2. Characterization of TVE-CuNPs

#### 3.2.1. UV-Vis Spectroscopy of TVE-CuNPs

**Figure 1:**
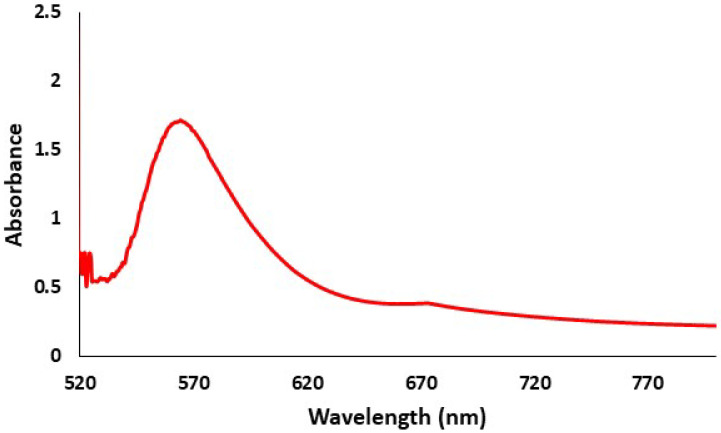
Biosynthesis of TVE-CuNPs using *Thymus vulgaris* extract.

After the formation of TVE-CuNPs, UV–Vis absorption was measured using a UV–visible spectrophotometer between 485 and 800 nm. Peaks at 573 nm were observed for pure TVE-CuNPs (Figure 1). Cu-NPs, in particular, generated noticeable absorption in the visible area in the range of 573–600 nm as a result of SPR.

#### 3.2.2. Size and Shape of TVE-CuNPs

**Figure 2:**
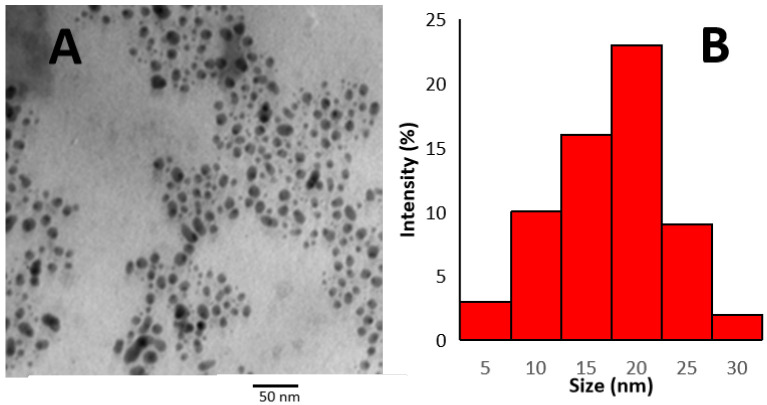
TEM images of the synthesized TVE-CuNPs (**A**) and percentage of the particle size distribution of TVE-CuNP (**B**).

TEM was used to investigate the TVE-CuNPs (Figure 2). TEM analysis determined the size of the synthesized TVE-CuNPs to be 10–25 nm. The particle size distribution of the TVE-CuNP picture further revealed that the TVE-AgNP size distribution ranged from 10 to 25 nm. TVE-CuNPs were spherical and monodispersed (Figure 2).

#### 3.2.3. X-ray Diffraction (XRD)

**Figure 3:**
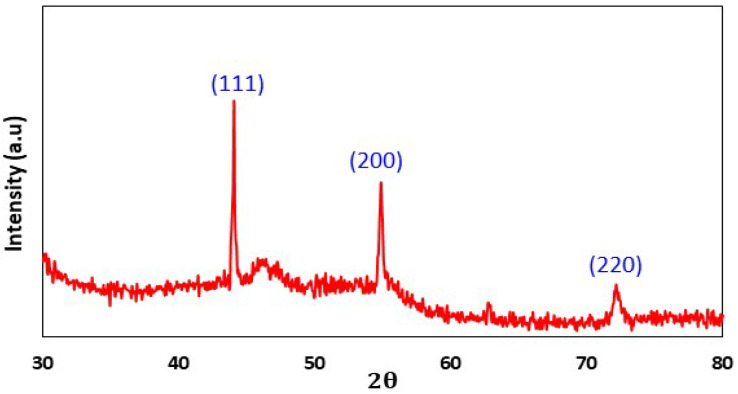
XRD patterns of the synthesized TVE-CuNPs.

The XRD pattern explained the sharp and distinct peaks of (2θ) 46.3°, 53.5°, and 72.3° that correspond to the planes (111), (200), and (220) of the pure copper’s face-centered cubic crystalline (FCC) structure (Figure 3). The purity of the TVE-CuNPs was reflected in the XRD results of this study.

#### 3.2.4. Comparison of FTIR Spectra of *Thymus vulgaris* Extract and TVE-CuNPs

**Figure 4:**
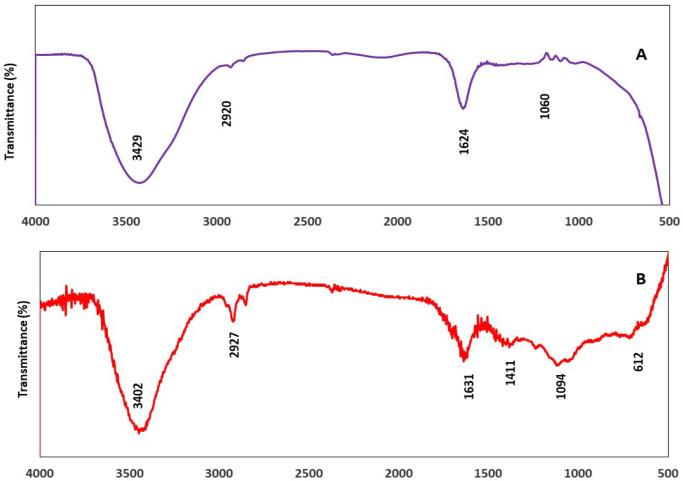
FTIR spectra of *Thymus vulgaris* extract (**A**) and the synthesized TVE-CuNPs (**B**).

FTIR analysis was used to characterize the TVE and TVE-CuNPs. As shown in Figure 4 and Table 1, the broad band at 3402 cm^−1^ is assigned to the O-H stretch of the polyphenol groups of *Thymus vulgaris* L extract. The peaks that appeared at 2934 cm^−1^ can be assigned to C–H in methyl and alkanes. The fewer absorption peaks are observed at regions of 1630 and 1421 cm^−1^, which belong to the C=O stretching of amide in the carbonyl stretch in proteins and enzymes. These proteins can be combined with metal nanoparticles via the carboxylate of amino acid remnants or free amine groups.

### 3.3. Characterization of AgNPs

**Figure 5:**
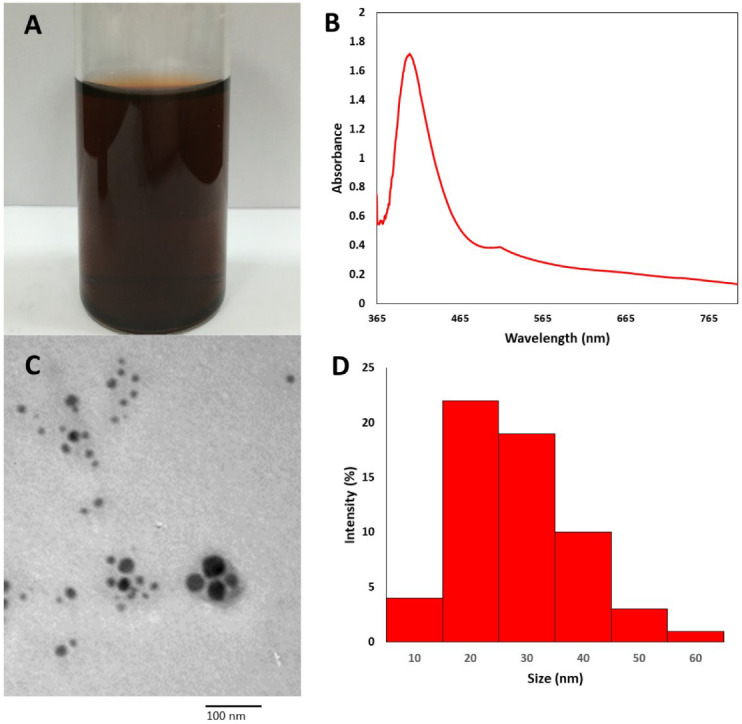
AgNPs characterizations: (**A**) AgNP solution; (**B**) UV–Vis spectrum absorption of the AgNPs; (**C**) AgNP TEM; and (**D**) AgNP size distribution.

The AgNPs (1 mg/mL) utilized in this investigation were obtained commercially. In deionized water, the solution was diluted to 100 µg/mL (Figure 5A). TEM and UV–Vis spectroscopy were used to examine the size, shape, and homogeneity of the AgNPs. The absorbance spectra revealed a single high peak at 405 nm (Figure 5B), indicating the existence of spherical AgNPs. TEM verified that the particles were spherical in form (Figure 5C). The AgNPs had an average diameter of 20–50 nm (Figure 5D).

### 3.4. Drug Release Determination

**Figure 6:**
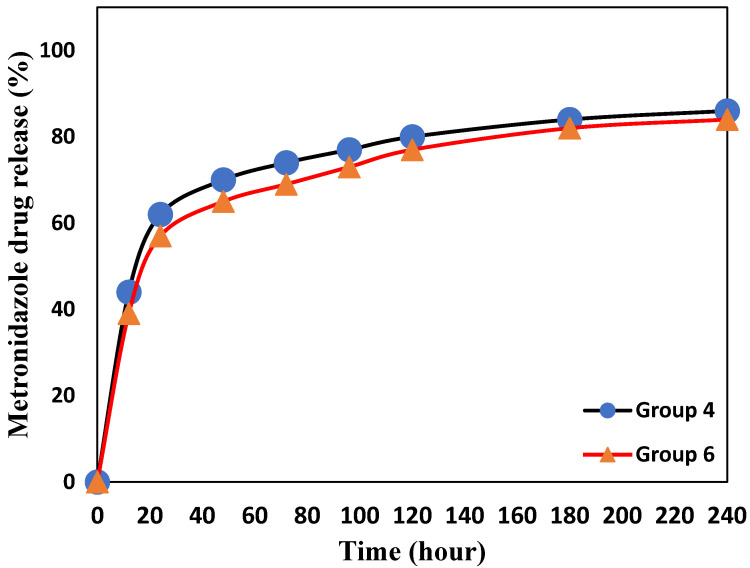
In vitro metronidazole release profile from GIC Group 4—1.5% metronidazole + 0.5% TVE-CuNPs + 98% GIC. Group 6—1.5% metronidazole + 0.5% AgNPs + 98% GIC.

Figure 6 depicts the metronidazole release (%) from the modified GIC groups C and D, revealing a two-step release: The quick first-step release, which is the metronidazole release (%) from GIC, in groups 4 and 6, reached 58% and 64% after 24 h, respectively. After 240 h, the delayed second-step release reached about 84% and 87%, respectively.

### 3.5. Antimicrobial Activity of the Tested Biomaterials

**Figure 7:**
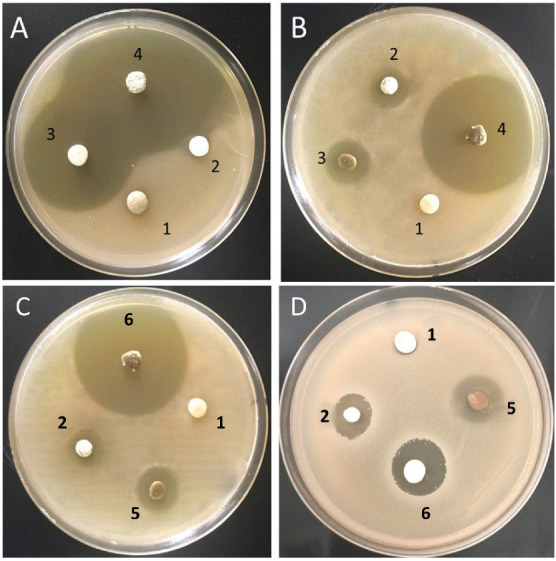
Antimicrobial activity of the biomaterials against *Staphylococcus aureus* (**A**,**C**) and *Streptococcus mutans* (**B**,**D**). 1—(Group 1) GIC; 2—(Group 2) 98.5% GIC with 1.5% metronidazole; 3—(Group 3) 99.5% GIC with 0.5% TVE-CuNPs; 4—(Group 4) 98% GIC with 0.5% TVE-CuNPs +1.5% metronidazole; 5—(Group 5) 99.5% GIC with 0.5% AgNPs; 6—(Group 6) 98% GIC with 0.5% TVE-CuNPs +1.5% metronidazole.

The results of agar disc diffusion assays against *S. mutans* and *S. aureus* revealed that groups 3 and 4 of GIC had a significant effect on bacterial growth inhibition when compared to other groups, as shown in Figure 7A,B. Furthermore, as shown in Figure 7C,D, GIC groups 5 and 6 inhibited bacterial growth significantly more than GIC groups 1 and 2. This effect was more pronounced when metronidazole and TVE-CuNPs or AgNPs were added, as in groups 4 and 6, which showed the statistically highest inhibition zones compared to groups 3 and 5, which contained TVE-CuNPs or AgNPs, (Group 4 > Group 3 > Group 2 > Group 1), as well as with GIC groups (Group 6 > Group 5 > Group 2 > Group 1), as shown in Table 2. The efficacy of GIC combined with metronidazole and TVE-CuNPs in group 4 was slightly lower than that of GIC combined with metronidazole and AgNPs in group 6. The inhibition zone and significant effects of metronidazole and TVE-CuNPs or AgNPs were the greatest in groups 4 and 6.

The mean inhibitory zones of the modified groups of GIC in the case of *S. aureus* were significantly higher than the mean inhibitory zones of these groups in the case of *S. mutans*. However, when compared to the other tested groups, groups 4 and 6 had the highest inhibition zones (see Figure 7 and Table 2).

**Figure 8:**
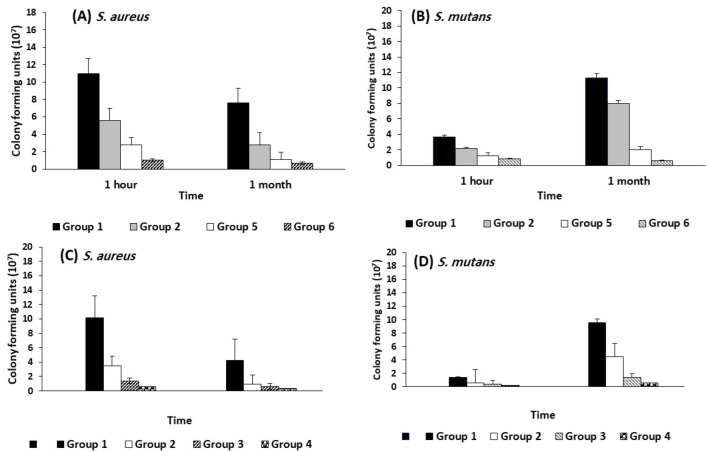
Number of CFU normalized to the control after one hour MDCT on the modified GIC cement and modified GIC cement after one month in suitable media (*n* = 3). 1—(Group 1) GIC; 2—(Group 2) 98.5% GIC with 1.5% metronidazole, 3—(Group 3) 99.5% GIC with 0.5% TVE-CuNPs; 4—(Group 4) 98% GIC with 0.5% TVE-CuNPs +1.5% metronidazole; 5—(Group 5) 99.5% GIC with 0.5% AgNPs; 6—(Group 6) 98% GIC with 0.5% TVE-CuNPs +1.5% metronidazole against *S. aureus* (**A**,**C**) and *S. mutans* (**B**,**D**).

Figure 8 depicts the MDCT assay results of GIC groups against *S. mutans* and *S. aureus*. The modified GIC (Group 4 > Group 3 > Group 2 > Group 1) and modified GIC (Group 6 > Group 5 > Group 2 > Group 1) had a significant antimicrobial effect against *S. mutans* and *S. aureus* when the antimicrobial effects of the GIC groups were compared. These were modified after one hour. In comparison to other groups, the modified GIC groups with AgNPs or TVE-CuNPs with metronidazole were more effective. At the end of the month, groups 4 and 6 had the highest antimicrobial efficacy against *S. aureus* and *S. mutans* when compared to the other groups (Figure 8), though the antimicrobial efficacy of groups 3 and 4 was slightly lower than that of groups 5 and 6, respectively, against *S. aureus* (as shown in Figure 8A,C) and *S. mutans* (as shown in Figure 8B,D). As a result, when compared to the other groups, GIC combined with antimicrobial agents (AgNPs or TVE-CuNPs and 1.5% metronidazole) demonstrated a superior antibacterial activity against *S. mutans* and *S. aureus*.

### 3.6. Compressive Strength Measurement

Table 3 shows the compressive strength of GIC in the following order: Group 3 ≥ Group 4 > Group 2 > Group 1 and Group 5 ≥ Group 6 > Group 2 > Group 1. Table 3 shows that, when GIC groups 4 and 6 were coupled with metronidazole and nano-copper or AgNPs, the compressive strength was slightly increased compared to GIC alone. Compressive strength measurements were not significantly different, indicating that the incorporation of metronidazole and/or nanoparticles did not reduce the strength of GIC for dental applications. The difference between all GIC groups was statistically insignificant (*p* > 0.05).

Currently, metronidazole and TVE-CuNPs or AgNPs with GIC are successfully biofabricated and exhibit the highest antibacterial efficiency against resistant *S. aureus* and *S. mutans* when compared to other modified materials (GIC with TVE-CUNPs or AgNPs). Because of the AgNPs effect, the modified GIC with metronidazole and TVE-CuNPs had a slightly lower antimicrobial efficiency than the GIC with metronidazole and AgNPs. However, both groups 4 and 6 show superior antimicrobial efficacy when compared to the other tested forms, which adds to the study’s novelty and significance in terms of controlling dental caries lesions.

## 4. Discussion

In this study, commercial AgNPs and TVE-CuNPs were synthesized using TVE and coupled with GIC with or without metronidazole to generate a new dental biomaterial. When GIC was mixed with the tested nanoparticles and metronidazole, its antibacterial effects were demonstrated when compared to GIC combined with AgNPs, TVE-CuNPs, or metronidazole alone. The current work validates the success of the biosynthesis of TVE-CuNPs from TVE based on our findings. The production of brown color in an aqueous solution of TVE-CuNPs was induced by surface plasmon resonance (SPR) excitation [21]. The production of TVE-CuNPs at the absorption band of around 573 nm due to SPR in TVE-CuNPs was verified by UV-Vis spectroscopy [22]. The TVE includes phytochemicals including phenol and protein amino acids, as well as enzymes that function as reducing and stabilizing agents for the greenly synthesized TVE-CuNPs and may also be responsible for the reduction of Cu+ to Cu0 [23]. TVE-CuNPs were spherical and monodispersed, with sizes ranging from 10 to 25 nm, and these results are in agreement with those of Suarez-Cerda et al. [24]. The TVE-CuNPs XRD pattern revealed three main peaks at 2θ values. The XRD pattern’s significant intensity and small breadth, and the sharpness of TVE-CuNP diffraction peaks indicated that the TVE-CuNPs were well crystalline. This study’s XRD data revealed the purity of TVE-CuNPs, which is similar to prior research [25,26]. The presence of large absorption peaks in TVE’s FTIR spectra proved the existence of reducing and stabilizing agents, such as proteins, amino acids, and phenolic chemicals (i.e., ellagic acid, thymol, -terpinene, -pinene, carvacrol) [23,25].

The AgNP characterization was undertaken. The spectra revealed a single high peak at 405 nm, indicating the presence of AgNPs, and TEM verified the particles’ spherical form [27]. Smaller nanosilvers have a peak at 400 nm, but bigger nanosilvers have higher scattering and expanded peaks pushed towards longer wavelengths, as observed in earlier research [27,28].

The drug release from GIC groups 4 and 6 was carried out in two stages. The primary rapid release during the first 24 h is very dependent on the strength of the drug attachment and might be caused by weak electrostatic interactions between metronidazole molecules and the GIC surface, hydrogen bond breakdown, and non-covalent connections [29]. The surface of GIC is susceptible to chemical and hydrolytic deterioration. Water can infiltrate the resin matrix and enhance matrix solubility, which is accelerated by a low pH [30]. As a result, the inorganic component of the GIC on the surface can dissolve and metronidazole can be released. For the next 216 h, a protracted and progressive release profile was seen. The release during this extended stage might be attributable to medication molecules trapped in the cement’s resin matrix. The two-step and prolonged release process is important in therapeutic treatments because the initial rapid release provides a therapeutic dosage rapidly and the subsequent long-term release maintains this dose over a long period of time. After 240 h, the released percentage was 86% of the total quantity.

The MDCT and agar disc diffusion assay results of GIC groups against *S. mutans* and *S. aureus* show that, when metronidazole and TVE-CuNPs or AgNPs were added, as in groups 4 and 6, there were statistically highest antimicrobial effect compared to groups 3 and 5, which contained TVE-CuNPs or AgNPs that were conjugated with SH groups in proteins and disrupted neuclic acids [6]. These findings are consistent with those of Mittal et al. [31] and Aguilar-Perez et al. [32], who found that the antimicrobial efficiency was affected by the concentration of metronidazole and nanoparticles used. Additionally, these findings are in agreement with the previous studies, which explain that the addition of nanoparticles to antibiotics enhances the antibacterial efficacy of clinically approved drugs [33] because metronidazole revives when mixed with TVE-CuNPs or AgNPs.

The findings of the GIC groups containing TVE-CuNPs were equivalent to those including commercial AgNPs against the tested bacteria, which exhibited somewhat decreased antibacterial activity, which was consistent with the findings of Zia et al. [34]. When compared to copper nanoparticles, silver nanoparticles were shown to be more reactive in inhibiting bacterial growth. According to Gad El-Rab et al. [35], *S. mutans* and *S. aureus* are very sensitive to nanoparticles containing antibiotics, which can be efficiently taken up by the bacteria because AgNPs and TVE-CuNPs regenerate the metronidazole to be active. Furthermore, AgNPs and TVE-CuNPs were conjugated with the SH group in proteins and damageed nucleic acids. In the current research, and for the first time, as shown in Table 4, GIC was combined with metronidazole and TVE-CuNPs or AgNPs, and the result demonstrates a significantly higher antimicrobial efficacy against the tested microbes when compared to the GIC with a single antimicrobial agent as well as the conventional GIC without any combinations [32,36,37]. Furthermore, the compressive strength difference across all GIC groups was statistically negligible (*p* > 0.05). These findings are consistent with those of Enan et al. [18], who discovered that adding nanoparticles to GIC did not result in a substantial improvement in compressive strength.

Currently, the combination of GIC, metronidazole, and AgNPs or TVE-CuNPs is successfully produced and exhibits high antimicrobial activity against the tested strains because metronidazole revives when mixed with TVE-CuNPs or AgNPs. It appears to have superior antimicrobial efficacy when compared to other forms. The GIC groups containing TVE-CuNPs were equivalent to those containing commercial AgNPs against the tested bacteria, although they exhibited somewhat decreased antibacterial activity. These findings add to the novelty and significance of the study that can be applied in the dental field to prevent dental caries. In the future, we will study the antimicrobial activity of these novel materials in vivo.

## 5. Conclusions

To conclude the results of the present study, *Thymus vulgaris* extract is a potential natural product for the green biosynthesis of TVE-CuNPs by the reduction of copper salts using reducing agents, such as phenolics and proteins, in addition to the enzymes present in TVE and for forming TVE-CuNPs. When metronidazole was combined with AgNPs or TVE-CuNPs, it was revived, and this combination with GIC formed novel nanobiomaterials. The GIC groups containing AgNPs exhibited somewhat increased antibacterial activity when compared with the GIC groups containing TVE-CuNPs. So, this material has superior antimicrobial activity against the resistant tested strains. Finally, this novel bio-material has potential to increase antimicrobial efficacy against the resistant tested strains and will result in the development of cost-effective dental materials for controlling bacterial infections and dental caries in the future.

## Figures and Tables

**Table 1:** Peaks were obtained by FTIR analysis and corresponding functional groups in *Thymus vulgaris* extract and TVE-CuNPs.

*Thymus vulgaris* Extract		TVE-CuNPs	
Peak (cm^−1^)	Functional Groups	Peak (cm^−1^)	Functional Groups
3429	Amine group (N–H) and the hydroxyl group (O–H)	3402	Amine group (N–H) and the hydroxyl group (O–H)
2920	C–H in methyl and alkanes	2927	C–H in methyl and alkanes
1624	C=O stretching of amide in carbonyl stretch in proteins/enzymes	1631	C=O stretching of amide in carbonyl stretch in proteins/enzymes
1060	C–O of proteins	1094	C–O of proteins

**Table 2:** Antimicrobial efficiency of modified dental luting cements against the tested bacteria.

Bacteria	Samples (Groups)	Inhibition Zone (mm)			ANOVA *F* Value	ANOVA *P* Value	Tukey’s Post Hoc
		1 Day	2 Weeks	1 Month			
*S. aureus*	a. Group 1	NA	NA	NA	NA	NA	NA
	b. Group 2	14 ± 1.62	13 ± 1.52	11 ± 0.57	7.62	0.02	1 day > 2 week and 1 month
	c. Group 3	20 ± 1.53	17 ± 1.21	15 ± 1.35	11.81	0.01	1 day > 2 week and 1 month
	d. Group 4	29 ± 1.11	22 ± 1.27	19 ± 1.56	13.43	0.001	1 day > 2 week and 1 month
	e. Group 5	24 ± 1.41	19 ± 0.57	16 ± 1.00	8.9	0.01	1 day > 2 week and 1 month
	f. Group 6	30 ± 1.71	23 ± 1.52	18 ± 1.15	14.30	0.001	1 day > 2 week and 1 month
	ANOVA F Value	16.71	14.65	17.35			
	ANOVA *p*-Value	0.0001	0.001	0.001			
	Tukey’s post Hoc	f > d > e > c > b	f > d > e > c > b	f > d > e > c > b			
*S. mutans*	a. Group 1	NA	NA	NA	NA	NA	NA
	b. Group 2	18 ± 1.93	16 ± 1.67	12 ± 1.58	9.62	0.03	1 day > 2 week and 1 month
	c. Group 3	19 ± 1.26	16 ± 1.31	13 ± 1.55	11.9	0.01	1 day > 2 week and 1 month
	d. Group 4	26 ± 1.34	21 ± 1.88	18 ± 1.17	16.30	0.001	1 day > 2 week and 1 month
	e. Group 5	20 ± 1.65	18 ± 1.53	14 ± 1.66	11.9	0.01	1 day > 2 week and 1 month
	f. Group 6	27 ± 1.35	23 ± 1.93	17 ± 1.69	16.30	0.001	1 day > 2 week and 1 month
	ANOVA F Value	16.26	15.52	14.36			
	ANOVA p-Value	0.001	0.001	0.001			
	Tukey’s post Hoc	f > d > e > c > b	f > d > e > c > b	f > d > e > c > b			

**1**—(Group 1) GIC; **2**—(Group 2) 98.5% GIC with 1.5% metronidazole; **3—**(Group 3) 99.5% GIC with 0.5% TVE-CuNPs; **4**—(Group 4) 98% GIC with 0.5% TVE-CuNPs +1.5% metronidazole; **5**—(Group 5)- 99.5% GIC with 0.5% AgNPs; **6**—(Group 6)-98% GIC with 0.5% TVE-CuNPs +1.5% metronidazole. All samples were analyzed in triplicate.

**Table 3:** Tested GIC samples’ compressive strength (MPa).

Tested Groups	*n*	Mean ± SD	ANOVA *F* Value	ANOVA *p* Value
Group 1	8	41.2 ± 3.4	2.74	0.165
Group 2	8	42.8 ± 2.8		
Group 3	8	44.2 ± 3.8		
Group 4	8	43.9 ± 3.7		
Group 5	8	45.9 ± 4.1		
Group 6	8	45.0 ± 4.5		

1—(Group 1) GIC; 2—(Group 2) 98.5% GIC with 1.5% metronidazole; 3—(Group 3) 99.5% GIC with 0.5% TVE-CuNPs; 4—(Group 4) 98% GIC with 0.5% TVE-CuNPs +1.5% metronidazole; 5—(Group 5) 99.5% GIC with 0.5% AgNPs; 6—(Group 6)-98% GIC with 0.5% TVE-CuNPs +1.5% metronidazole.

**Table 4:** Comparison of earlier research with restorative biomaterials for antibacterial activity against oral bacteria.

Materials	Concentration	Bacteria	Reference
GIC + CuNPs	96% GIC + 4% CuNPs	*S. mutans* and *S. sanguinis*	Aguilar-Perez et al. [32]
GIC + antibiotics	98% GIC + 2% antibiotic	*S. mutans*	Singer et al. [36]
GIC + AgNPs	GIC + 0.5% AgNPs	*S. aureus*, *S. mutans* and *C. albicans*	Ashour et al. [37]
GIC + TVE-CuNPs + metronidazole	98% GIC + 0.5% TVE-CuNPs + 1.5% metronidazole	*S. aureus*, and *S. mutans*	This study
GIC + AgNPs + metronidazole	98% GIC + 0.5% AgNPs + 1.5% metronidazole	*S. aureus*, and *S. mutans*	This study

## Data Availability

Data will be made available upon request from the corresponding author.

## Sample Availability

Samples of the compounds are not available from the authors.
